# β-Hydroxybutyrate mitigates the detrimental effects of high glucose in human retinal pigment epithelial ARPE-19 cells

**DOI:** 10.1007/s13577-025-01187-x

**Published:** 2025-02-20

**Authors:** Francesca Argentino, Marta Mallardo, Ciro Costagliola, Aurora Daniele, Ersilia Nigro

**Affiliations:** 1 CEINGE-Biotecnologie Avanzate Scarl “Franco Salvatore”, Via G. Salvatore 486, 80145 Naples, Italy; 2 International School of Advanced Studies, Center for Neuroscience, University of Camerino, 62032 Camerino, MC Italy; 3 Department of Molecular Medicine and Medical Biotechnology, University of Naples “Federico II”, Via Pansini 5, 80131 Naples, Italy; 4 Department of Neurosciences, Reproductive Sciences and Dentistry, Eye Clinic, University of Naples “Federico II”, Naples, Italy; 5 Department of Environmental, Biological and Pharmaceutical Sciences and Technologies, University of Campania “Luigi Vanvitelli”, Via Vivaldi 43, 81100 Caserta, Italy

**Keywords:** Hyperglycemia, β-Hydroxybutyrate, Human retinal pigment epithelial ARPE-19 cells, Inflammation

## Abstract

High glucose leads to cellular damage and dysfunction in the retina. Dietary interventions, including the use of ketogenic diets, have been explored for their potential to reduce the adverse effects of hyperglycemia. β-Hydroxybutyrate (BHB), a ketone body, has immune and anti-inflammatory properties. This study aims to investigate whether BHB ameliorates the harmful effects induced by high glucose in ARPE-19 cells, a model of retinal pigment epithelium. We investigated the effects induced by high glucose and/or BHB on viability, migration, colony-forming ability, cell cycle progression and cytokine production. Our data indicate that high glucose significantly reduces the viability of ARPE-19 cells with no significant changes in apoptosis or autophagy, while inducing cell cytostasis. On the other hand, BHB exerts a protective effect on ARPE-19 cells under hyperglycemic conditions improving cell viability and alleviating glucose-induced cell cycle arrest. Additionally, BHB treatment affects the expression of IL-8 and IL-17α, as well as of MCP-1, modulating the inflammatory response, cell migration and wound healing. In conclusion, this study highlights the potential protective role of BHB against the detrimental effects induced by high glucose on ARPE-19 cells. These findings support the use of ketone bodies in mitigating high glucose-induced cellular damage. Future research will be critical to translate these findings to the clinical practice for metabolic diseases.

**Supplementary Information:**

The online version contains supplementary material available at 10.1007/s13577-025-01187-x.

## Introduction

Diabetic retinopathy (DR) is a major complication of diabetes mellitus and a leading cause of blindness in the working-age population worldwide [1]. The pathogenesis of DR is multifactorial, involving hyperglycemia-induced oxidative stress, inflammation, and neurodegeneration within the retinal microenvironment [2]. One of the key cellular targets in DR is the retinal pigment epithelium (RPE), a monolayer of highly specialized cells that play a crucial role in maintaining retinal homeostasis and visual function [3]. ARPE-19 cells, a human RPE cell line, are widely used as an in vitro model to study the effects of hyperglycemia and other stressors on retinal health [4].

Previous studies have reported glucose-induced oxidative stress and subsequent cell cycle arrest in retinal cells [5–7]. Moreover, hyperglycemia-induced oxidative stress can cause mitochondrial dysfunction characterized by a decrease in mitochondrial membrane potential and ATP production, contributing to cellular energy deficits and impaired cell proliferation [8].

These cellular responses contribute to the progression of DR by impairing the regenerative capacity of the RPE and promoting chronic inflammation [2]. The interplay between oxidative stress, mitochondrial dysfunction, and cellular senescence highlights the complex nature of hyperglycemia-induced retinal damage and underscores the need for therapeutic strategies targeting multiple pathways.

Recent studies have also highlighted the significance of cytokines in the pathophysiology of DR [9]. During proliferation arrest, senescence cells are metabolically active and are more likely to secrete inflammatory mediators such as interleukins. IL-8, IL-17 and monocyte chemoattractant protein-1 (MCP-1) are cytokines typically produced by RPE cells [10, 11] that have been implicated in several pathological processes in the retina. IL-8 is a pro-inflammatory cytokine known to recruit neutrophils and other immune cells to sites of inflammation [12]. It has been shown to play a critical role in mediating inflammatory responses under hyperglycemic conditions, contributing to the chronic inflammation observed in DR [1]. Elevated levels of IL-8 found in the vitreous fluid of DR patients further support its involvement in the inflammatory aspect of the disease [13]. IL-17 is another cytokine typically involved in inflammation [14]; previous studies reported its role in inflammatory mechanisms in retinal cells and DR cellular models [15]. MCP-1, on the other hand, is a key chemokine involved in the recruitment of monocytes and macrophages, which has also been associated with the regulation of cell migration and proliferation [16]. Studies have demonstrated that MCP-1 can influence the migration of cells, promoting wound healing and colony formation, processes that are essential for tissue repair and regeneration [17].

Recent research has highlighted the role of dietary interventions in managing diabetes and its complications [18, 19]. Specifically, ketogenic diets, which are high in fats and low in carbohydrates, have gained attention for their ability to induce ketosis and produce high levels of ketone bodies, such as β-Hydroxybutyrate (BHB). BHB has emerged as a potential therapeutic agent, because it is not only an alternative energy source but also exhibits neuroprotective and anti-inflammatory properties [20]. Studies have shown that ketogenic diets and BHB can mitigate oxidative stress and improve cellular viability under various stress conditions [21, 22]. In the context of DR, BHB’s ability to counteract hyperglycemia-induced damage in retinal cells holds promise for novel therapeutic interventions [23, 24].

The present study aims to investigate the in vitro protective effects of BHB on ARPE-19 cells under hyperglycemic conditions. In detail, we examined the viability, migration, and colony-forming ability of ARPE-19 cells exposed to high glucose levels with or without BHB co-treatment. Additionally, we explored the molecular mechanisms underlying these effects by analyzing cell cycle progression, apoptosis, and the expression of key cytokines, such as IL-8, IL-17 and MCP-1, known to be involved in inflammatory processes of the retina.

Understanding the interplay between diet, metabolic health, and cellular responses in DR is crucial for developing comprehensive treatment strategies. By elucidating the protective mechanisms of BHB in hyperglycemia-induced damage, we hope to provide insights into potential dietary interventions that could complement existing therapies for DR.

## Materials and methods

### Cell culture

The human ARPE-19 cell line was kindly provided by the Bank of Human and Animal Continuous Cell Lines-CEINGE Biotecnologie Avanzate “Franco Salvatore”, Napoli, Italy. Cells were cultured in Dulbecco’s medium (DMEM) (Sigma-Aldrich, MO, United States) supplemented with 10% of heat-inactivated Fetal Bovine Serum (FBS) (Sigma-Aldrich, MO, United States) and 1% l-glutamine (Sigma-Aldrich, MO, USA). Cells were grown in a 5% CO_2_ humidified incubator, at 37 °C. To choose the optimal doses for the experiments, dose–response curves were performed for both glucose (Sigma-Aldrich, MO, USA) and BHB by treating the cells with increasing concentrations of the compounds (glucose: 5.5 mM, 50 mM, 100 mM, 200 mM, 250 mM; BHB: 1 mM, 5 mM). All experiments were performed in 5% FBS medium. Glucose was diluted in distilled water to obtain a 1 M stock; BHB was diluted in distilled water to obtain a 0.5 M stock.

### Cell viability assay

Cell viability was assessed using the 3-[4,5-dimethylthiazol-2-yl]−2,5-diphenyltetrazolium bromide (MTT) colorimetric assay as previously reported [25]. Briefly, ARPE-19 cells (4 × 10^3^ cells/well) were seeded in 96-well plates. The following day, the cells were treated with glucose (200 mM), BHB (5 mM) or co-treated with both. After 24 and 48 h of treatment, the cells were stained with 5 mg/mL of MTT reagent for approximately 4 h. The formazan crystals that were formed were further dissolved in 100 μL of DMSO and the absorbance was measured at 550 nm using a microplate reader (Model 550, Ultramar Microplate Reader; Bio-Rad, CA, United States). The final readings were compared with the untreated control samples. The experiments were performed three times in triplicate. Additionally, cell viability was also measured using the trypan blue reagent (Bio-Rad, CA, USA). Briefly, cells (2 × 10^6^) were seeded in 6-well plates. The following day, cells were treated with glucose (200 mM), BHB (5 mM) or co-treated with both compounds. After 24 and 48 h, cells were harvested and counted using the TC10TM Automated Cell Counter (Bio-Rad, CA, United Staes). The experiments were performed twice in duplicate.

### LDH release assay

The lactate dehydrogenase (LDH) assay was used for the quantification of cell death and cell lysis. ARPE-19 cells were seeded into 6-well plates at a density of 4 × 10^4^ cells/well. The day after, the cells were subjected to treatment with glucose (200 mM) and/or BHB (5 mM) for 24 and 48 h. Subsequently, each supernatant (100 μL) was combined with 100 μL of the reaction mixture (0.7 mM INT; 54.0 mM lactic acid; 0.3 mM phenazine methosulfate; and 0.8 mM NAD + in 0.2 M Tris–HCl pH 8.0) in a 96-well plate. The reaction proceeded for 45 min in darkness under gentle stirring at 37 °C and was halted by the addition of 1 M HCl (50 μL). Absorbance readings were taken at 490 nm using a Tecan Spectra Fluorescence and absorbance reader. The data acquired were normalized with respect to a blank prepared using a Triton X-100 (1%) solution. The detected enzyme activity in the culture supernatant mirrors membrane integrity and correlates with the proportion of lysed cells.

### Cell cycle analysis

ARPE-19 cells were plated and allowed to grow overnight to 70–80% confluency. The cells were treated with glucose and/or BHB for 24 and 48 h. The cells were then collected and resuspended in 0.3 mL of pre-cooled 1 × phosphate-buffered saline (PBS) followed by 0.7 mL of −20 °C ethanol. They were left on ice for a minimum of 30 min and finally pelleted via centrifugation, resuspended in a 1 × PBS solution of 2.5 mg/mL propidium iodide (Sigma-Aldrich; St. Louis, MO, USA), RNAse (1 mg/mL) (Sigma-Aldrich; St. Louis, MO, USA) and 0.1% Triton X-100 in PBS and incubated for 1 h before a flow cytometry analysis (BD LSRII; Becton Dickinson, San Diego, CA, USA).

### Wound healing assay

ARPE-19 cells were seeded in a 6-well plate at the concentration of 3 × 10^5^/well in complete culture media and grown to confluence. The day after, cells were treated with 4 μg/ml of mitomycin (Sigma-Aldrich) for 2 h to inhibit cell proliferation, and then a wound was inflicted using a tip. After washing with PBS, cells were incubated with glucose 200 mM, BHB 5 mM, or the combination of the two in comparison to untreated cells. The same positions along the scratch wound were observed and photographed immediately after the wound (0 h) and at different time points (18 h, 24 h and 30 h) using an inverted-phase-contrast microscope (Nikon microscope TS100 fluorescence and video camera). The rate of wound closure was calculated with the software ImageJ and expressed as percentage of the closure.

### Colony formation assay

ARPE-19 cells were seeded in 6-well plates at the concentration of 1 × 10^3^ cells/well. After 24 h, cells were treated with glucose 200 mM and/or BHB 5 mM and allowed to grow for seven days. The growth medium was replaced every 3 days. After 7-day incubation, these plates were washed with PBS twice, fixed by 4% paraformaldehyde (PFA) (Invitrogen, CA) for 30 min and colored in 1% crystal violet solution for 20 min and washed repeatedly in water. Colonies were counted manually using a light microscope as previously described [26]. Experiments were performed two times in triplicate.

### Western blot assay

The total protein content was extracted from the cells using a pre-cooled radioimmunoprecipitation assay (RIPA) buffer containing a protease inhibitor cocktail. Later, the protein content was quantified via Bradford’s method. Successively, the samples were diluted in Laemmli buffer 4 × and boiled for 5 min at 95 °C. Then, 30 to 40 μg of total cellular proteins was loaded into a polyacrylamide gel and separated via SDS-PAGE. Thereafter, the proteins were transferred to PVDF membranes, blocked with 5% non-fat milk and then incubated at 4 °C overnight with the primary antibodies, according to the manufacturer’s instructions: Ulk-1, ubiquitin, beclin, GAPDH, APAF1, LC3A/B. The day after, the membranes were incubated with anti-mouse or anti-rabbit antibodies coupled to horseradish peroxidase. Finally, protein bands were detected via a Chemi Doc XRS (Bio-Rad, Hercules, CA, USA), using ECL detection reagents (Pierce Biotechnology, Waltham, MA, USA). To visualize multiple proteins on the same blot, the blots were cut and/or stripped using a stripping solution (Bio-Rad, Hercules, CA, USA), followed by re-incubation with specific primary antibodies.

### RNA extraction and quantitative real time-PCR

Total RNA was isolated from ARPE-19 cells using the TRIzol Reagent (Thermo Fisher Scientific, Waltham, MA, USA). The RNA concentration was determined through fluorescence-based detection with a Qubit 4 Fluorometer (Thermo Fisher Scientific, Waltham, MA, USA). One microgram of total RNA was converted to cDNA using the SuperScript III First-Strand Synthesis SuperMix (Thermo Fisher Scientific, Waltham, MA, USA), following the manufacturer’s protocol. Gene expression analysis was conducted on a C1000 Touch Thermal Cycler (Bio-Rad, Hercules, CA, USA) using iQ SYBR Green Supermix (Bio-Rad, Hercules, CA, USA). The thermal cycling conditions were as follows: an initial denaturation at 95 °C for 3 min, followed by 40 cycles of 95 °C for 10 s, 60 °C for 30 s, and 72 °C for 30 s. GAPDH was utilized as the housekeeping gene, and fold changes in gene expression were calculated using the 2^−ΔΔCt^ method. The primer sequences used for qRT-PCR are available upon request.

### Statistical analysis

Data are expressed as mean of replicates ± standard deviation (SD). GraphPad Prism 6 software (GraphPad Software, California, United States) was used to carry out the analyses. Statistical comparisons between the control and treatments were performed using the one-way or two-way ANOVA followed by the Tukey multiple comparisons test. A p value < 0.05 was considered statistically significant.

## Results

### High glucose impairs ARPE-19 Cell viability, but the co-treatment with BHB partially restores this effect

**Fig. 1:**
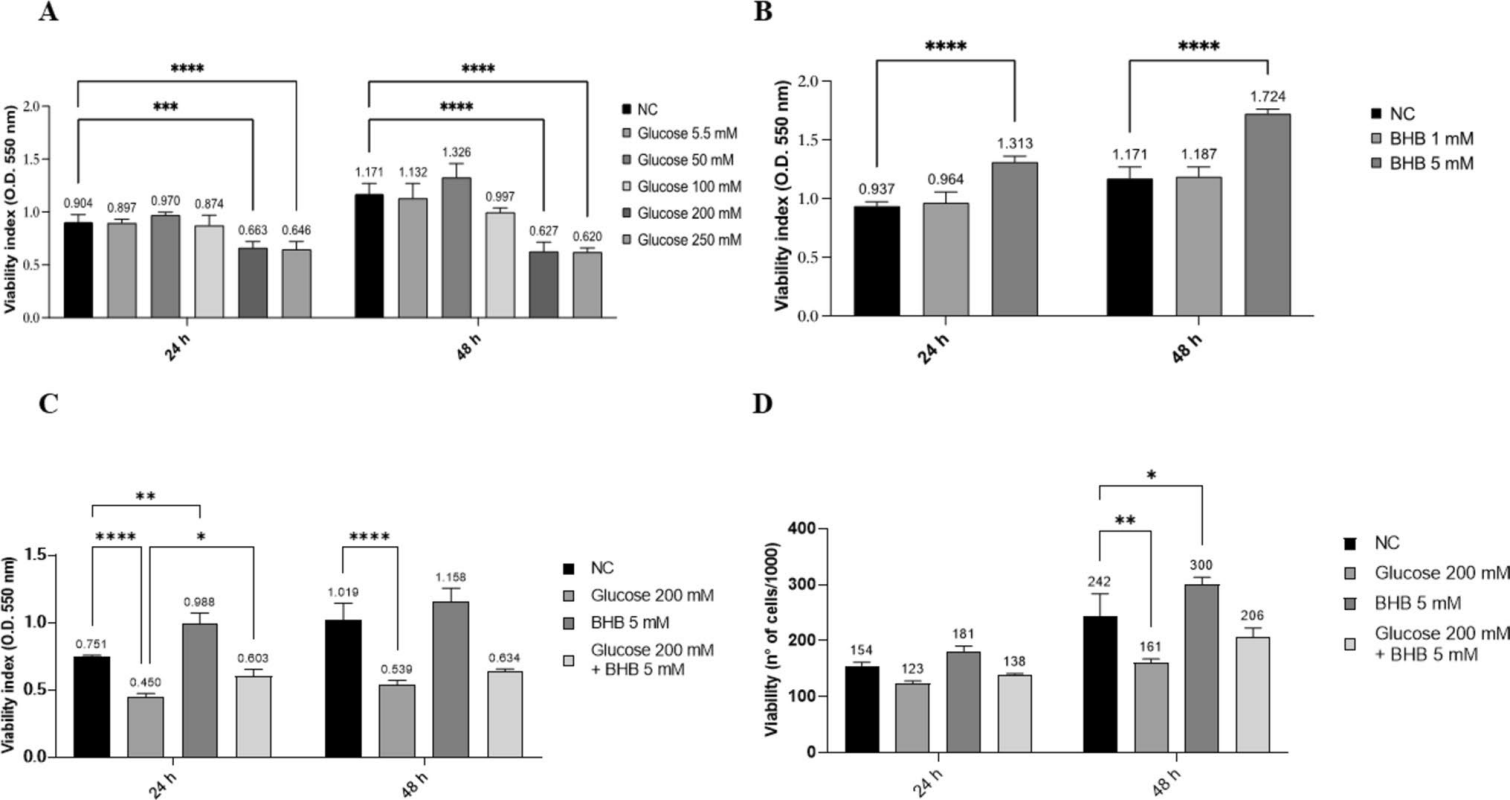
Reduction in cell viability caused by high glucose conditions is partially reverted by BHB. **a**, **b** Dose/response curves to determine the optimal doses of glucose and BHB. The selected doses were 200 mM for glucose and 5 mM for BHB. **c** Cell viability was assessed via an MTT assay after 24 and 48 h of treatment with glucose 200 mM and/or BHB 5 mM. The experiments were performed three times in triplicate. **d** The percentage of viability was also calculated using the trypan blue assay after 24 and 48 h of the treatments reported above. Values are expressed as the mean of two different experiments performed in duplicate ± SD. * *p* < 0.05; ** *p* < 0.01; *** *p* < 0.001; **** *p* < 0.0001

The impact of glucose and BHB on ARPE-19 cell viability was assessed. We established the doses of glucose and BHB after performing a dose/response curve: the selected concentrations were 200 mM for glucose and 5 mM for BHB (Fig. 1A, B), to respectively mimic hyperglycemic and ketogenic conditions. ARPE-19 cells, maintained in a 5% FBS medium, were left untreated (negative control: NC) or treated with 200 mM glucose and/or 5 mM BHB for 24 and 48 h, followed by an MTT assay. As expected, high concentrations of glucose significantly reduced the viability of ARPE-19 cells (*p* < 0.01). Interestingly, BHB significantly improved cell viability when used alone and in combination with high concentrations of glucose, compared to untreated and high glucose conditions, respectively (Fig. 1C). Additionally, we assessed cell viability by counting the vital cells in the different experimental conditions. The data confirmed the detrimental effects of high glucose on cell viability and the ability of BHB to partially restore them (Fig. 1D). Further in accordance, cell cycle analysis reveals an increase in the percentage of cells in the G2 phase at 24 h when cultured under high glucose conditions compared to untreated cells, indicating cell cycle arrest. Conversely, cells supplemented with BHB exhibited a doubling of the S phase compared with the control at both 24 and 48 h, a finding reflected also in the cell count. To explore the molecular mechanisms behind glucose-induced cell toxicity and BHB partial reversion, we conducted an LDH release assay. The assay showed no significant differences in LDH levels across the four conditions, suggesting that glucose did not cause cell lysis (Supplementary Fig. 1).

**Fig. 2:**
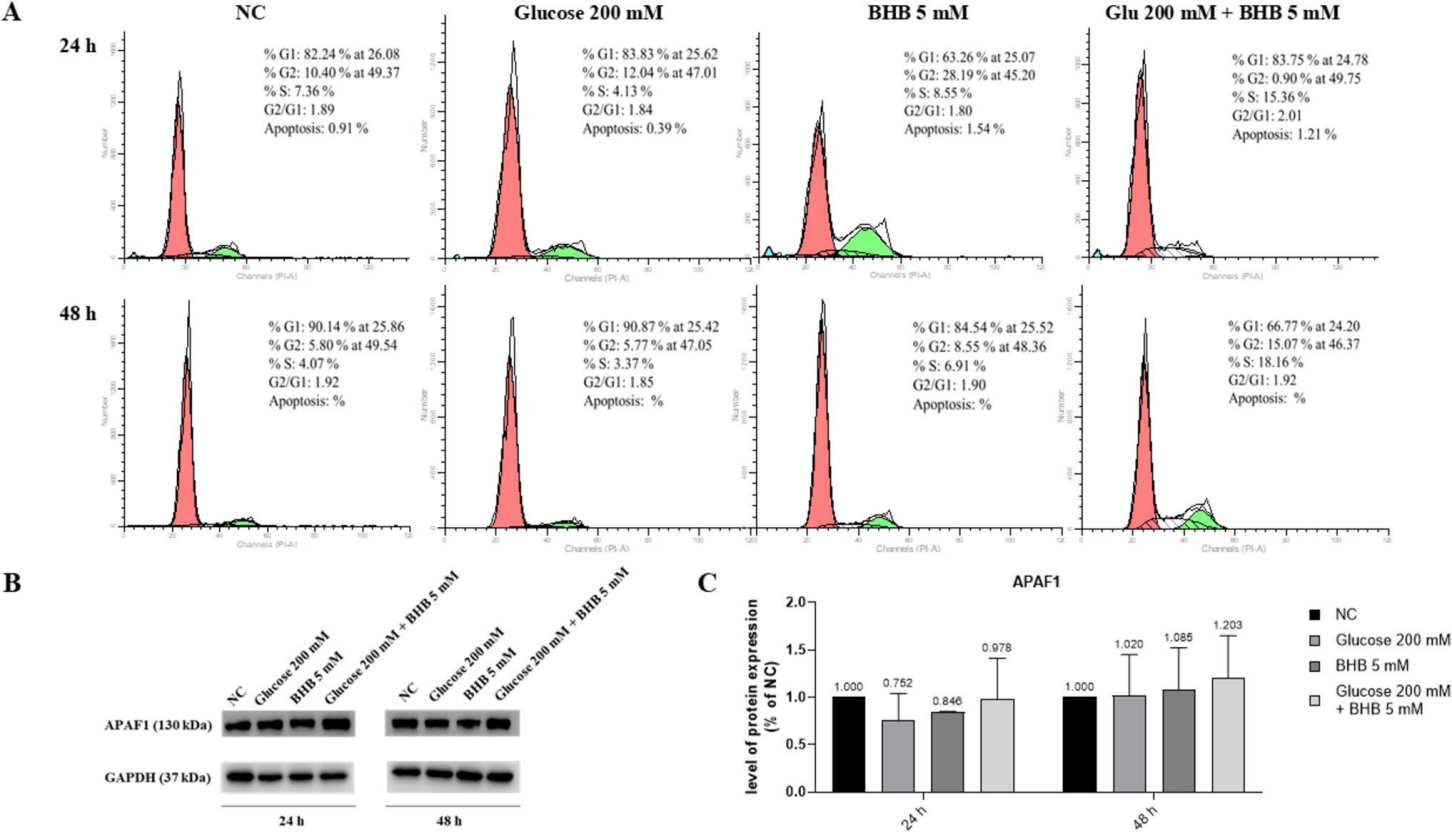
High glucose-induced slow-down of cell cycle division is partially reverted by the co-treatment with BHB. **a** A cell cycle analysis of ARPE-19 cells treated with glucose 200 mM and/or BHB 5 mM for 24 and 48 h is shown. Representative FACS histograms of the ARPE-19 cells exposed or not (NC) to the treatments are reported. The experiment was performed twice. **b** A representative Western blot image of APAF1 and its respective GAPDH, which was used as internal loading control. **c** Graphical representation of the densitometric analysis performed to normalize the data to GAPDH

We therefore investigated the possibility that apoptosis or autophagy may be implicated. In particular, we analyzed the cell cycle to evaluate the effects of high glucose and/or BHB on the progression. Cell cycle was analyzed through flow cytometry after 24 and 48 h of exposure to 200 mM glucose and/or 5 mM BHB. Figure 2A illustrates that high glucose concentrations negatively impacted the ARPE-19 cell cycle, whereas co-treatment with BHB resulted in an increase in the percentage of cells in the S phase, by 11.23% at 24 h and 14.79% at 48 h. An increase in cells in the S phase indicates a higher percentage of cell proliferation. Cells treated solely with BHB also showed enhanced cell cycle progression (7.36% NC vs. 8.55% BHB at 24 h; 3.37% NC vs. 6.91% BHB at 48 h). These findings suggest that high glucose slows down ARPE-19 cell cycle division at 24 and 48 h, while BHB promotes an increase in the S phase, promoting cell proliferation. To further explore the apoptotic flux of ARPE-19 cells, we performed a Western blot analysis of APAF-1 protein. The experiments showed no significative differences in the levels of the protein expression among the different experimental conditions, further confirming the absence of apoptosis involvement (Fig. 2B, C).

**Fig. 3:**
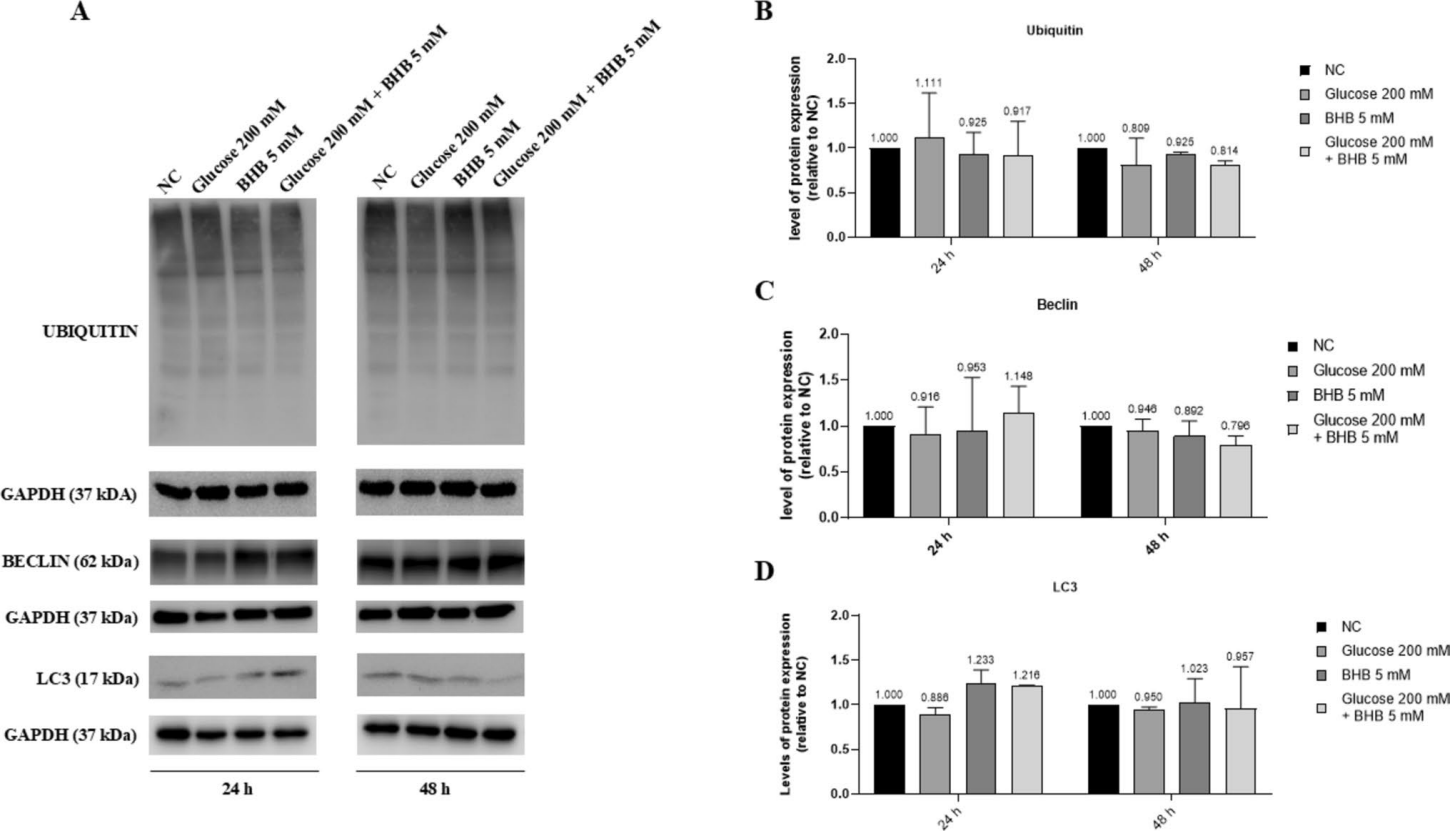
Glucose toxicity does not induce a modulation in two proteins associated with autophagy in ARPE-19 cells. Western blot analyses were carried out using GAPDH as internal loading control; a densitometric analysis was performed to normalize the data. **a** A representative Western blot image of ubiquitin, beclin, LC3A/B and their respective GAPDH. **b**,** c**,** d** Graphical representations of pixel quantization of ubiquitin, beclin and LC3A/B, respectively, normalized to their GAPDH. Values are expressed as mean of three different experiments ± SD. NC: untreated cells. The statistical analysis was evaluated using the two-way ANOVA test

At this point, we also conducted a Western blot of some proteins associated with autophagy, that revealed that high glucose and BHB as well do not change the expressions of Ulk-1 (data not shown), ubiquitin, beclin and LC3A/B in ARPE-19 cells (Fig. 3). The western blot analysis also showed no cleavage of LC3A/B protein, a mechanism typically associated with autophagy.

### BHB partially restores the impaired capability of wound healing induced by high glucose in ARPE-19 cells

**Fig. 4:**
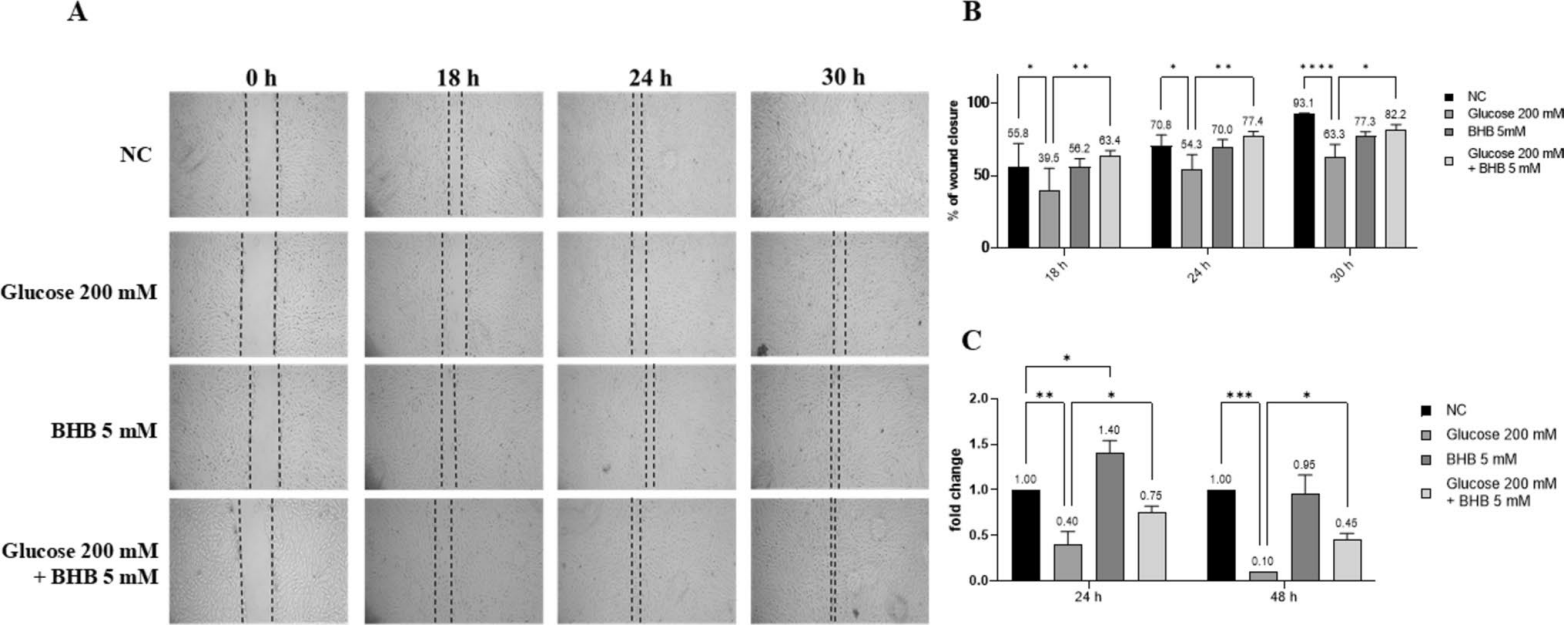
High glucose hampers cell migration, while the co-treatment with BHB mitigates this effect. A scraped wound was inflicted on a confluent monolayer of ARPE-19 cells, that were then treated with glucose 200 mM, BHB 5 mM or a combination of both. **a** Cells were photographed under microscopy at different time points (0 h, 18 h, 24 h and 30 h). **b** The wound’s area was calculated measuring the length of the scratch in pixels and expressed as percentages of wound closure. **c** Expression levels of MCP-1 mRNA were determined via a q-PCR; the data were standardized using GAPDH as internal control and then quantified via the 2^−ΔΔCt^ method. The experiments were performed twice in triplicate. * *p* < 0.05; ** *p* < 0.01; *** *p* < 0.001; **** *p* < 0.0001

The effect of high glucose and BHB on ARPE-19 cell migration, a key biological event in DR, was evaluated using the wound healing assay. Specifically, as illustrated in Fig. 4, high glucose significantly inhibited cell migration compared to untreated cells (*p* < 0.05). While BHB alone did not significantly alter the wound healing capability, it partially restored the cell migration ability of ARPE-19 cells when used in combination with glucose (*p* < 0.05, *p* < 0.01). To further investigate this aspect, we used a RT-qPCR to examine the expression of MCP-1, a known chemoattractant expressed by ARPE-19 cells. The results showed an increase in its expression elicited by BHB, both alone (*p* < 0.05) and in co-treatment with glucose (*p* < 0.05), indicating its involvement in cell migration (Fig. 4C).

### High glucose-induced impairment of colony formation ability is reverted by co-treatment with BHB in ARPE-19 cells

**Fig. 5:**
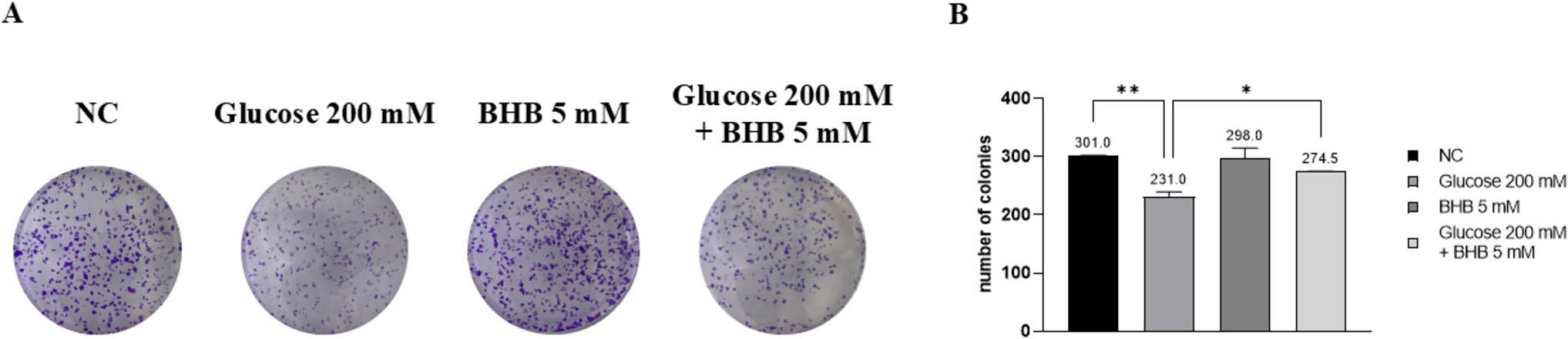
High glucose impairs the ability to form colonies, while co-treatment with BHB partially reverts this effect. **a** Cells were fixated, stained and photographed after seven days from treatment. The experiment was performed twice in duplicate. **b** Colonies were counted manually. * *p* < 0.05; ** *p* < 0.01

Colony ability was evaluated in cells treated with both glucose and BHB. ARPE-19 cells were exposed to 200 mM glucose, 5 mM BHB, or a combination of both for 7 days and subsequently tested for colony formation (Fig. 5A, B). The data suggest a significant restoration of the colony-forming capability in ARPE-19 cells co-treated with 5 mM BHB and 200 mM glucose, compared to cells treated solely with glucose (*p* < 0.05).

### The induction of high glucose of IL-8 release is mitigated by BHB in ARPE-19 cells

**Fig. 6:**
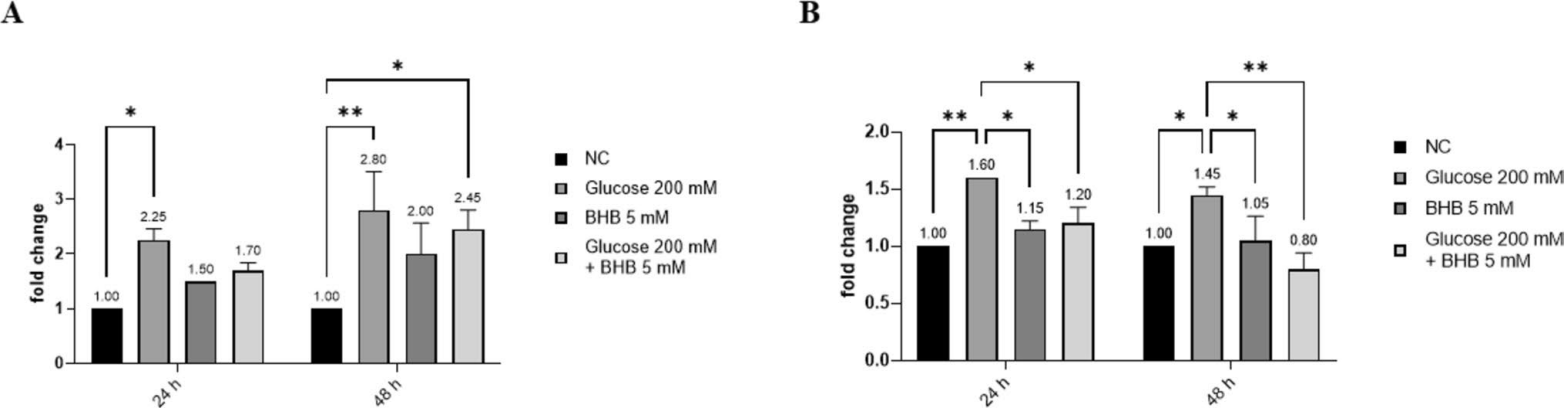
The high glucose-induced higher expression of pro-inflammatory cytokines is mitigated by the co-treatment with BHB. mRNA levels were determined via a q-PCR; the data were standardized using GAPDH as internal control and then quantified via the 2^−ΔΔCt^ method. **a** Expression levels of IL-8. **b** Expression levels of IL-17. The experiments were performed twice in triplicate. * *p* < 0.05; ** *p* < 0.01

Our next investigation regarded the effects of high concentrations of glucose on inflammatory cytokines, including IL-6, IL-8, IL-17 and IL-10 and whether the co-treatment with BHB could revert them. Using a RT-qPCR we observed that, while there were no significant changes in the expression of IL-6, IL-10 and TNF-α through the analyzed conditions (data not shown), IL-8 and IL-17 levels were significantly increased when ARPE-19 were treated with high glucose. These are known pro-inflammatory cytokines, typically produced by retinal cells [27, 28]. Interestingly, the co-treatment with BHB 5 mM significantly reverted this detrimental effect for IL-17 (*p* < 0.05), and resulted in a reducing trend of the expression of IL-8 (Fig. 6).

## Discussion

The present study aimed to investigate the effects of high concentrations of glucose on ARPE-19 cells taken as an in vitro model of DR and to evaluate the potential protective role of BHB, a ketone body typically produced during ketogenic diet regimens. Hyperglycemia, a hallmark of diabetes, exerts several detrimental effects on retinal cells, including oxidative stress, inflammation, and metabolic dysregulation [29]. These effects contribute to the pathogenesis of DR by impairing retinal cell function and survival [30].

Our data demonstrate that high glucose concentrations significantly reduce the viability of ARPE-19 cells without increasing LDH levels, suggesting a cytostatic rather than a cytotoxic effect. Interestingly, our analysis showed no significant changes in the levels of proteins associated with autophagy and did not reveal an apoptotic induction by high glucose, reinforcing the notion that glucose-induced cytostasis in ARPE-19 cells does not involve these cell death pathways. In agreement with our data, previous findings have demonstrated that high glucose levels can induce a senescent-like state in retinal cells, characterized by oxidative stress and cell cycle arrest rather than apoptosis [31].

BHB, a ketone body produced during states of ketosis, has garnered attention for its potential neuroprotective and cytoprotective effects [32]. In our study, BHB supplementation had a protective effect on ARPE-19 cells under high glucose conditions by supporting cell proliferation and mitigating the cytostatic effects induced by high glucose levels.

The protective effects of BHB observed in this study may be attributed to its ability to serve as an alternative energy source thereby supporting cellular metabolism and reducing oxidative stress [33]. Studies have shown that BHB can activate protective signaling pathways, which may explain its beneficial effects in hyperglycemic conditions and its modulation of repair mechanisms [22, 34]. In fact, BHB has been shown to modulate several epigenetic pathways, thus leading to the upregulation of genes involved in cell survival, metabolism, and stress response, thereby contributing to its cytoprotective effects [34]. In addition, high glucose significantly impairs ARPE-19 cell migration, consistent with earlier reports indicating that hyperglycemic conditions hamper wound healing in diabetes [35]. BHB co-treatment partially restored the migratory ability of these cells, indicating its potential role in promoting retinal repair. This is significant because impaired cell migration can hinder tissue repair and regeneration processes, particularly in the retina where the migration of cells plays a critical role in maintaining retinal function and angiogenesis [36].

The colony formation assay provided further insight into the long-term effects of high glucose and BHB on ARPE-19 cells. High glucose conditions severely diminished the colony-forming capability of these cells, which is indicative of reduced proliferative potential and cellular senescence [31]. However, co-treatment with BHB enhanced the colony-forming ability of ARPE-19 cells compared to glucose treatment alone, highlighting its potential role in supporting cell survival, but also in promoting long-term proliferation and tissue-regeneration. Such findings are supported by literature suggesting that ketone bodies can stimulate cellular growth and repair mechanisms [20].

Additionally, inflammation plays a pivotal role in DR, through the activation of the resident microglial cells in the retina [37]. For this reason, we investigated the cytokine production in ARPE-19 cells in high glucose conditions and with or without the co-treatment with BHB. IL-8 is known to be one of the most typical cytokines produced by retinal pigment epithelial (RPE) cells, playing crucial roles in the inflammatory response and chemotaxis [27, 38]. Nonetheless, IL-17 is also involved in these mechanisms and its pro-inflammatory effects are well studied both in in vivo and in vitro studies of retinal pathologies [39, 40]. Using RT-qPCR, we observed that high glucose led to an upregulation of their expression: this aligns with existing literature that highlights the inflammatory responses elicited by glucose toxicity in RPE cells [41]. The co-treatment with BHB, however, showed a modulation in the expression levels of this cytokines, suggesting a potential anti-inflammatory role of BHB.

Another important chemokine produced by RPE cells is MCP-1 [42], which functions as a chemoattractant and an activator for lymphocytes and monocytes. It was found in previous studies that MCP-1 levels increased during the first phases of wound healing [43, 44], and our analysis confirmed the involvement of this chemokine in cell migration. In fact, the RT-qPCR revealed higher expression levels of MCP-1 in the ARPE-19 cells treated with BHB with respect to the untreated cells, as well as increased expression in the co-treatment of high glucose and BHB with respect to the cells only treated with high concentration of glucose.

The observed alterations in the cytokine levels in our cells are relatively relevant, even considering the absence of specific inhibitors, which could have provided more precise insights into the mechanistic role of this cytokines. Nonetheless, these findings contribute to the growing body of evidence highlighting the cytokines’ role in DR and other inflammatory diseases, supporting their involvement in the pathological mechanisms.

While BHB’s metabolic benefits are well-documented, its influence on cellular behaviors and in the context of DR has not been extensively explored. Interestingly, our findings are supported by recent studies highlighting the role of BHB in promoting cellular functions beyond its metabolic impact. For instance, a study on SW480 colon cancer cells treated with 5-fluorouracil (5FU) demonstrated that BHB promotes proliferation, migration, and stemness, indicating significant metabolic plasticity in these cells [45]. This study suggests that BHB can enhance cell survival and function, which aligns with our observations in ARPE-19 cells. Recent findings also investigated the beneficial effects of this ketone body on neural health, highlighting its potential role in neuroinflammation and neuroprotection [46].

Further research should explore the detailed molecular mechanisms by which BHB exerts these protective effects, nevertheless the findings in our study suggest its potential therapeutic application in DR and other retinal diseases characterized by hyperglycemia-induced damage. The importance of healthy dietary choices is gaining increasing attention in recent years, including and especially in relation to various pathological conditions. In the context of DR, given the close relationship of its pathological mechanisms with elevated glycemic levels, ketogenic diets and ketone bodies represent promising strategies. Traditional treatments for DR, such as laser photocoagulation and anti-VEGF therapy, primarily focus on managing the symptoms rather than addressing the underlying cellular dysfunction. BHB, by enhancing cellular resilience, offers a novel approach to protect retinal cells and potentially slow the progression of DR. Moreover, BHB’s ability to support cellular survival may have broader implications for other neurodegenerative and metabolic diseases. Conditions such as Alzheimer’s disease, Parkinson’s disease, and amyotrophic lateral sclerosis (ALS) involve mitochondrial dysfunction and oxidative stress, similar to DR. The neuroprotective effects of BHB observed in preclinical studies highlight its potential as a therapeutic agent across a diverse range of diseases [20, 47].

Our findings contribute to the growing body of literature on the protective effects of BHB and ketone bodies on health. By restoring cell viability, migration, and colony formation, BHB demonstrates potential therapeutic benefits for mitigating the effects of DR. However, to fully realize the therapeutic potential of ketogenic diets and ketone bodies, further research is needed to elucidate the molecular mechanisms and optimize the delivery for clinical applications. Preclinical studies should focus on understanding the long-term effects of BHB treatment, its pharmacokinetics, and potential side effects. Additionally, clinical trials are necessary to evaluate the efficacy and safety of BHB in patients with DR and other retinal diseases.

## Conclusions

In conclusion, this study highlights the detrimental effects of high glucose on ARPE-19 cells and the potential protective role of BHB. These findings contribute to the growing body of evidence supporting the use of ketone bodies in mitigating hyperglycemia-induced cellular damage and pave the way for future therapeutic developments in DR and potentially other neurodegenerative and metabolic diseases. Future research will be critical in translating these findings into clinical practice, with the potential to improve outcomes for patients suffering from these debilitating conditions.

## Supplementary Information

Below is the link to the electronic supplementary material.

Supplementary file1 (DOCX 88 KB)

Supplementary file2 (TIF 68 KB)

## Data Availability

The data will be made available upon request.
