# Supplementary material for: β-Hydroxybutyrate mitigates the detrimental effects of high glucose in human retinal pigment epithelial ARPE-19 cells
Source: Hum Cell. 2025 Feb 20;38(2):59. doi: 10.1007/s13577-025-01187-x (PMC11842486; doi:10.1007/s13577-025-01187-x)
Supplement: Supplementary file 1 — Supplementary file1 (DOCX 88 KB) [file 13577_2025_1187_MOESM1_ESM.docx]

**
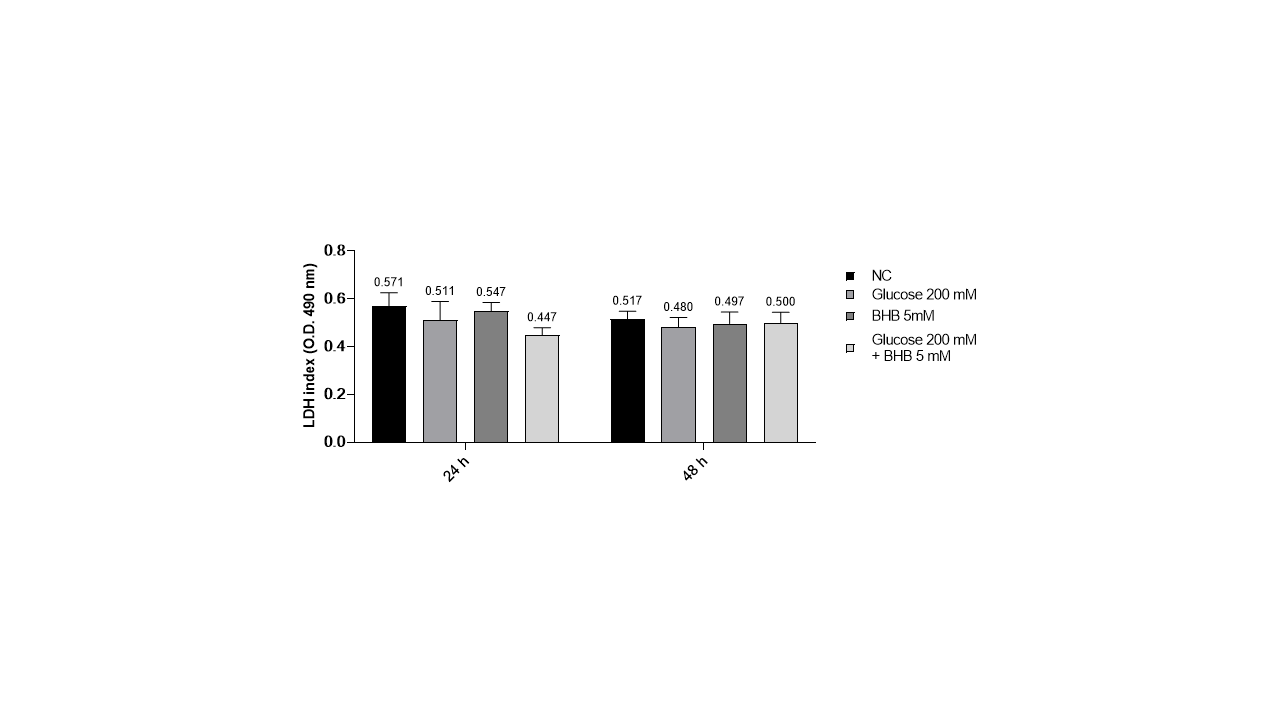
**

**Supplementary Fig. 1** High glucose conditions do not induce cell lysis in ARPE-19 cells. A LDH release assay was performed to evaluate the lysed cells in the different experimental conditions.
