# Supplementary figures and images for: β-Hydroxybutyrate mitigates the detrimental effects of high glucose in human retinal pigment epithelial ARPE-19 cells
Source: Hum Cell. 2025 Feb 20;38(2):59. doi: 10.1007/s13577-025-01187-x (PMC11842486; doi:10.1007/s13577-025-01187-x)

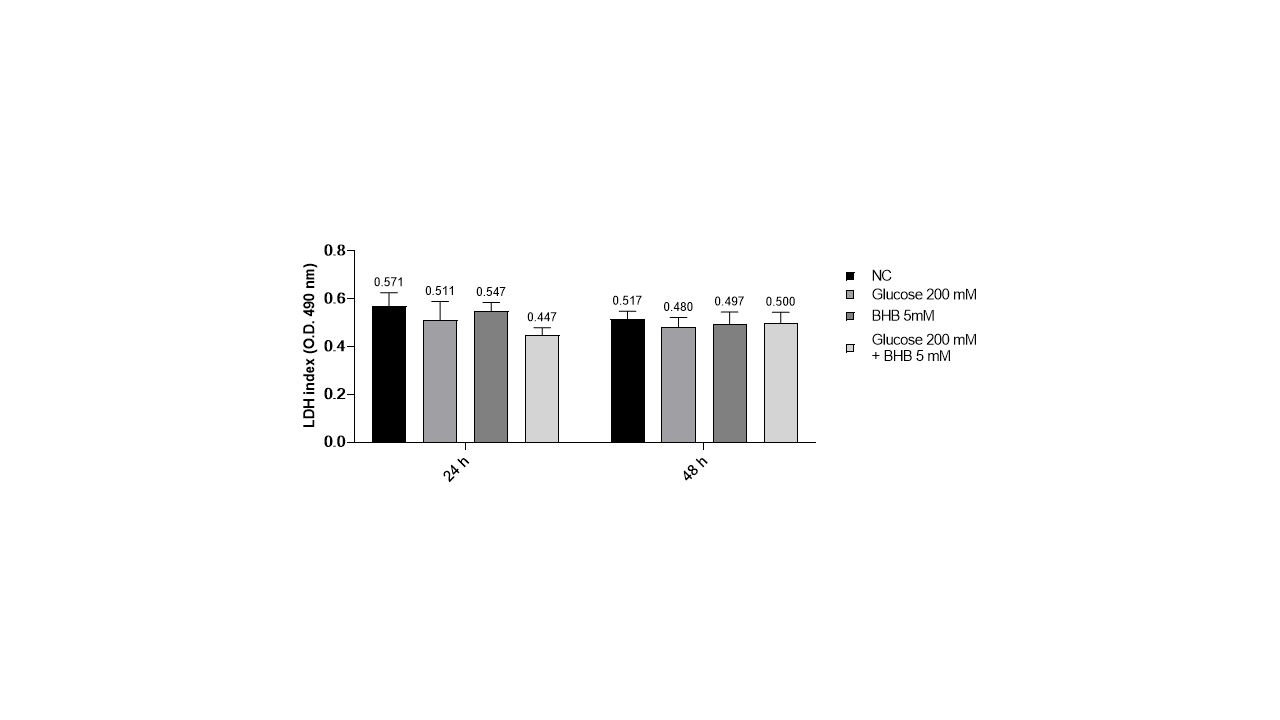

Supplement: Supplementary file 2 — Supplementary file2 (TIF 68 KB) [file 13577_2025_1187_MOESM2_ESM.tif]
